# 

**Published:** 1857-06

**Authors:** 


					﻿PUBLISHED MONTHLY, AT 83 PER ANNUM IN ADVANCE.
. ...........
T' L JV K T TV
WEDICAL AND SVBDICAL S
' W » »	1:
P:DITED BY
JOSEPH F. LOGAN, M. D.,	|
PSOFEBSOK OF PHTSIOLOOT AXD UeXSRAL PaTHOLOOT,
'<
W. F. WESTMORELAND, M. D.,
------------— /
Pax et Bcieatia, led veritas sine timore.	/
__-----------------
f t
■	■	r
Vol. n.	JUNE, 1857.	No. 10. 6
. ....
ATLANTA, GEORGIA:
F»TtINTBI> BY O- F». EDDY Ab CO.	/
(Successors to)	5
C. K. HANLEITER k GQ.
1857.	§
tS" Each Number contains sixty-four pages. Postage.—Under 800 miles, 15 cents a year;	t
over SOf' nines. 30 cents a year—to be pre-paid quari.erly. If not pre-paid, double tha
above rates.	,
				

